# Molarization of Mandibular Second Premolar

**DOI:** 10.5005/jp-journals-10005-1251

**Published:** 2014-08-29

**Authors:** Neha Mangla, Vineet Inder Singh Khinda, Shiminder Kallar, Gurlal Singh Brar

**Affiliations:** Postgraduate Student, Department of Pedodontics and Preventive Dentistry Genesis Institute of Dental Sciences and Research, Ferozepur Punjab, India; Professor and Head, Department of Pedodontics and Preventive Dentistry Genesis Institute of Dental Sciences and Research, Ferozepur Punjab, India; Reader, Department of Pedodontics and Preventive Dentistry Genesis Institute of Dental Sciences and Research, Ferozepur Punjab, India; Senior Lecturer, Department of Pedodontics and Preventive Dentistry Genesis Institute of Dental Sciences and Research, Ferozepur Punjab, India

**Keywords:** Macrodontia, Mandibular second premolar, Molarization

## Abstract

Macrodontia (megadontia, megalodontia, mac rodontism) is a rare shape anomaly that has been used to describe dental gigantism. Mandibular second premolars show an elevated variability of crown morphology, as are its eruptive potential and final position in the dental arch. To date, only eight cases of isolated macrodontia of second premolars have been reported in the literature. This case report presents clinical and radiographic findings of unusual and rare case of isolated unilateral molarization of left mandibular second premolar.

**How to cite this article:** Mangla N, Khinda VIS, Kallar S, Brar GS. Molarization of Mandibular Second Premolar. Int J Clin Pediatr Dent 2014;7(2):137-139.

## INTRODUCTION

Dental organogenesis disorders manifest as alterations in the number, size or form of teeth.^1^ When dental size and anatomy present characteristics that deviate from what is supposed to be accepted range of normality, they are termed anomalies.^2^ Mandibular second premolars show an elevated variability of crown morphology, as are its eruptive potential and final position in the dental arch.^3^

Macrodontia (megadontia, megalodontia, mac rodontism), is a rare shape anomaly that has been used to describe dental gigantism.^4,5^ O’Sullivan^6^ reported the prevalence of macrodontia to be 1 to 2% in males and 0.9% in females, but macrodontia of mandibular second premolars affected males and females equally. Canoglu^4^ et al reported an overall prevalence of macrodont permanent teeth to be 0.03 to 1.9% with a higher frequency in males. In most of the cases, macrodontia in mandibular second premolars has been reported in children.^7,8^ All the reported unilateral cases of macrodontia involved the right mandibular second premolar.^3^

Macrodontia is usually associated with systemic disturbances or syndromes, such as insulin-resistant diabetes, otodental syndrome, facial hemihyperplasia, KBG syndrome, Ekman-Westborg-Julin syndrome and 47 XYY syndrome.^4,5^ Isolated form of macrodontia has rarely been reported.^4,9^ To date, only eight cases of isolated macrodontia of second premolars have been reported in the literature; five of which have shown bilateral occurrence.^1,4^ No case report of unilateral erupted macrodont mandibular second premolar has been reported in the literature.

This case report presents clinical and radiographic findings of unusual and rare case of isolated unilateral molarization of left mandibular second premolar.

## CASE REPORT

**Fig. 1:**
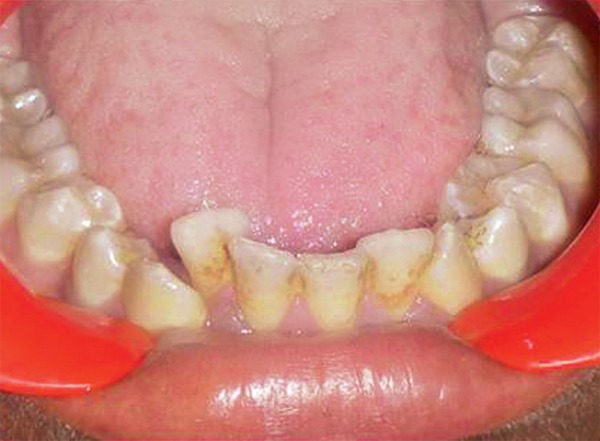
Intraoral preoperative photograph

**Fig. 2:**
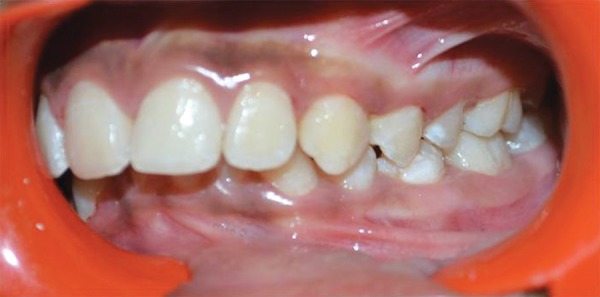
Occlusion of the patient with Angle’s class I molar relation

A 14-year-old male patient reported to our clinic for routine dental check-up. There was no relevant family and medical history elicited. Extraoral examination revealed no abnormalities. On intraoral examination, dental caries was present involving the buccal pits of permanent mandibular right and left first and second molars. The patient had extrinsic stains and calculus with respect to mandibular anterior region (Fig. 1). He presented with Angle’s class I molar relation (Fig. 2).

**Fig. 3:**
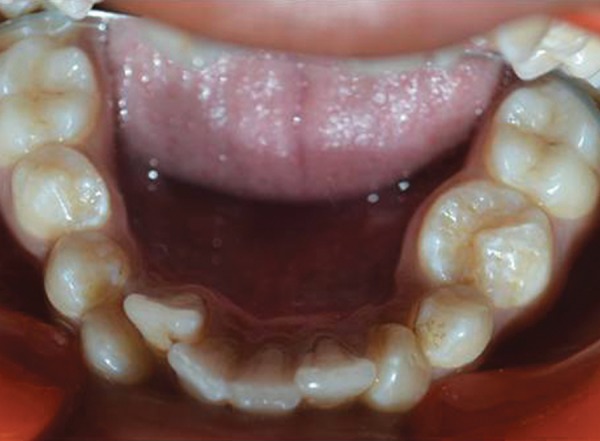
Molariform appearance of mandibular left second premolar

**Fig. 4:**
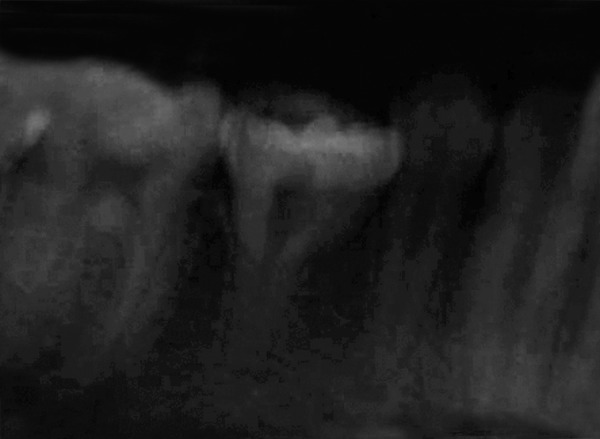
Intraoral periapical radiograph of the patient depicting unusual morphology of mandibular left second premolar

**Fig. 5:**
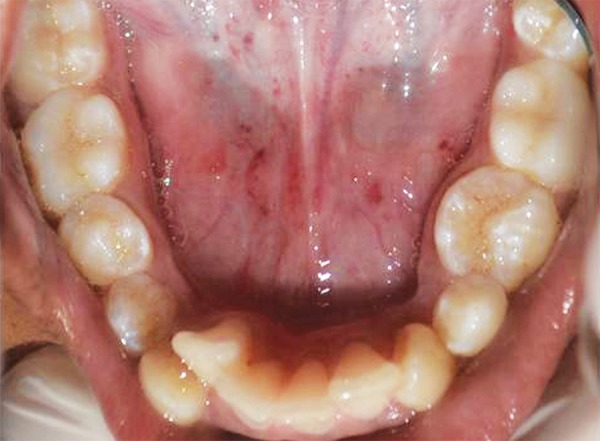
Postoperative photograph of the patient

The mandibular left second premolar had an abnormal ovoid molariform crown with an irregular crescent-shaped crater-like fissure with the convex aspect toward the lingual side, in occlusion (fully erupted) which resulted in severe crowding in mandibular anterior region (Fig. 3). Clinically, mandibular left second premolar presented with mesiodistal diameter of 11 mm and buccolingual diameter of 11.5 mm. To determine the diagnosis of the anomaly, intraoral periapical radiograph was taken. Radiographically, tooth represented with an abnormal size and shape with a single tapering root of normal length (Fig. 4). Oral prophylaxis was done, and composite restorations were done for the cariously involved teeth (Fig. 5). Patient was referred for fixed orthodontic therapy.

## DISCUSSION

The molar-like morphology of the premolars consists of a reduction of the single vestibular cusp, the shoulders of which appear as small extra cusps. The resulting appearance is the same as that of a mandibular first molar, with three vestibular cusps and three, two, one or no lingual cusps. In studies of dental anthropology and hominid evolution, descriptions are found, such as that of *Australopithecus robustus*, in which the premolars are shaped like molars, with large occlusal surfaces and one, two or three roots.^3^ The etiology of dental anomalies remains largely unclear but some anomalies in tooth structure, shape and size results by many factors from disorders during the morphodifferentiation stage of development. Identification of specific patterns of associated dental problems could be related with certain genetic and environmental factors contributing to different dental anomaly subphenotypes.^7^

Macrodontia can be classified as true generalized where teeth are larger than normal and are associated with pituitary gigantism, relative generalized where there is presence of normal or slightly larger than normal teeth in small jaws and macrodontia involving single tooth.^10,11^ True macrodontia of a single tooth should not be confused with fusion of teeth, in which early in odontogenesis, the union of two or more teeth results in a single large tooth.^10^

According to the classification of macrodontia, this case corresponds to an isolated macrodontia. It is uncommon to see localized macrodontia alone, because generally it is associated with a syndrome.^7^ This type of macrodontia is more frequently found in incisors and canines,^5,8,12^ and has been rarely reported to involve premolars and molars. All the reported unilateral cases of macrodont mandibular second premolars demonstrate the involvement of the right tooth but, in the present case, the left tooth was involved.^8^

The mesiodistal (11 mm) and buccolingual diameter (11.5 mm) of mandibular second premolar was greater than its normal size of 7 and 8 mm respectively.^13^ Thus, the appearance of severe crowding was a predictable consequence of the increased size of the mandibular second premolar. Under normal conditions, the mesiodistal size of the premolar is less than that of its deciduous predecessors, particularly in the case of mandibular second premolars.^3,14,15^ Problem of plaque accumulation is found in such cases because of surface notching as in this reported case.^16^

The large crown size causes problems with eruption and disrupts the dentition. There are consequent inherent difficulties for the extraction of these teeth. Once erupted, their anatomy predisposes them to caries.^8^

## CONCLUSION

The dental anomaly of unilateral macrodontia of mandibular second premolar appears to be extremely rare. Dental professionals should acquire deeper knowledge about this anomaly and carry out careful treatment planning to avoid unexpected problems during dental treatment procedures generated by the ignorance of morphology.
